# Effects of postpartum hormonal changes on the immune system and their role in recovery

**DOI:** 10.3389/abp.2025.14241

**Published:** 2025-06-11

**Authors:** Xueqin Wu, Rong Jin

**Affiliations:** Department of Obstetrics and Gynaecology, The People’s Hospital of Danyang, Affiliated Danyang Hospital of Nantong University, Danyang, China

**Keywords:** postpartum, mother recovery, hormonal changes, immune system, inflammatory reactions

## Abstract

**Background:**

The postpartum period involves complex physiological changes, notably in hormone levels, that significantly influence immune system function. Hormonal regulation during pregnancy prevents maternal immune rejection of the fetus, but following childbirth, these hormone levels drop rapidly, leading to immune reconstitution.

**Aim:**

This review investigates the impact of hormonal changes on immune system dynamics during the postpartum period and highlights their implications for maternal recovery.

**Methods:**

The study analyzed current literature, focusing on hormonal influences, particularly cortisol, prolactin, estrogen, and progesterone, on immune reconstitution with associated inflammatory responses in the postpartum period.

**Results:**

Postpartum immune reactivation, triggered by hormonal shifts, can lead to a resurgence of inflammatory reactions. This process, characterized by increased cortisol and prolactin levels and a rapid decline in estrogen and progesterone, could exacerbate dormant autoimmune conditions or trigger latent infections, making this period especially vulnerable to immune-related complications.

**Conclusion:**

Hormonal and immune responses are closely interdependent in the postpartum period, leading to heightened susceptibility to infections, autoimmune flare-ups, and other immune-related disorders. For improved postpartum care and enhanced maternal health outcomes, more research is necessary to clarify the mechanism of immune reconstitution, find possible hormonal indicators, and create focused therapeutic approaches. This review further highlights the critical role of hormonal-immune crosstalk in postpartum mood disorders (PPD, postpartum anxiety [PPA], and postpartum psychosis [PP]), proposing integrated biomarkers for early intervention.

## Introduction

Most women will, at some point in their lives, become pregnant. Pregnancy has an effect on nearly every bodily function. Pregnancy causes significant and pervasive physiological changes that help the unborn child develop and be cared for later (Zhang et al., 2023a). Women in the postpartum period may experience postpartum depression (PPD), while the majority of new moms will experience the baby blues, a temporary mood disturbance that occurs early in the postpartum period (Dazzan et al., 2022). In contrast to the baby blues, these conditions are more severe and stay longer, frequently enduring for months or even a year. Numerous psychosocial risk factors that overlap between PPD and PPA include stress exposure during pregnancy, prenatal or past history of anxiety and depression, and more (Costello et al., 2023). Furthermore, mood swings, irritability, restlessness, changes in appetite, exhaustion, and cognitive deficits are some of the symptoms that PPD and PPA could exhibit in common. The elevated rate of comorbidity between PPD and PPA is a result of these consistency issues (Bogovié Crněié et al., 2023). Extreme melancholy, hopelessness, and anhedonia, or disinterest, are characteristics of PPD. This lack of interest frequently affects the infant as well, which can negatively impact the mother-infant attachment and the mother’s capacity to care for the child (Harding and Heaton, 2022).

**FIGURE 1:**
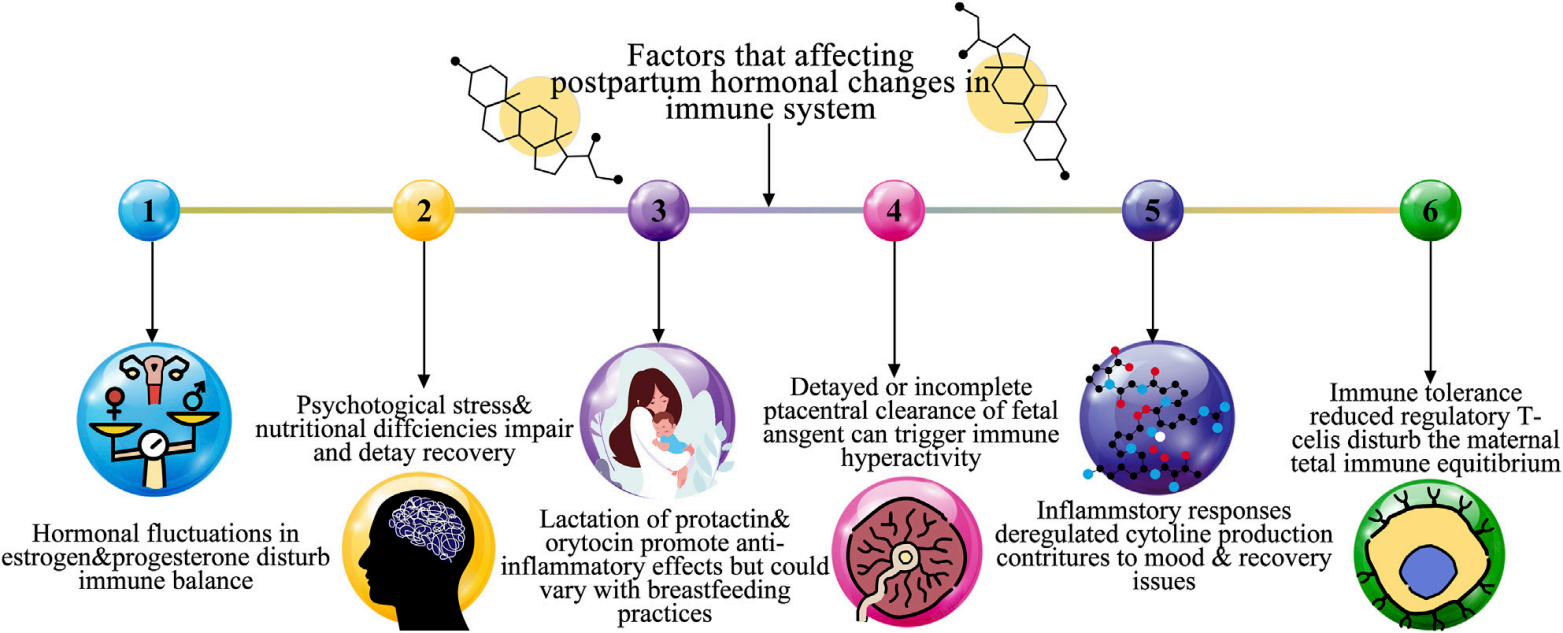
Overall factors affecting a postpartum hormonal change in immune system.

Although PPA typically presents as anxiety and panic focused on the child, it can also affect maternal care and cause behavioral abnormalities like unnecessary inspection on or share the infant as a way to counteract the obsessive thought (Peng and Pearce, 2022). But women with an individual or relations history of PP, schizoaffective disorder, are at a considerably greater possibility of developing PP (Saharoy et al., 2023). PPD, the majority harsh of the after giving birth intellectual disorder, is typified by disassociation from reality, hallucinations, and delusions. Due to the high rates of infanticide and suicide, PP poses a significant risk to the safety of both mother and child (Neumann et al., 2024). The prevalence, symptoms, risk factors, and effects of postpartum mental illnesses are highlighted in Table 1. Although baby blues are normal and temporary, PPD, PPA, and Postpartum psychosis (PP)are serious health hazards for both the mother and the child that call for focused treatments. Figure 1 represents the overall factors that affect the postpartum hormonal and immune system.

**TABLE 1:** Postpartum mental health conditions and their contributing elements.

Disorder	Prevalence (%)	Key symptoms	Risk factors	Potential impact
Postpartum Anxiety (PPA)	10–20	Overwhelming anxiety, panic episodes, and compulsive holding and Checking	Overlap with risk factors for PPD	Disturbances in the development of infants and maternal care
Postpartum psychosis (PP)	0.1 (1 in 1000 births)	Hallucinations, delusions, paranoia, and detachment from reality	Schizoaffective disorder and bipolar disorder, family history	Extreme danger to the mother’s and child’s safety
Baby Blues	85	Erratic moods, irritation, restlessness, fatigue, and short-term	Changes in hormones after delivery	Few long-term consequences
Postpartum depression (PPD)	10–20	Extreme melancholy, despair, anhedonia, and cognitive impairments	Anxiety and a past history of anxiety or depression	Deteriorated maternal care with bond to the mother

The common of investigations based on hormonal and neuronal pathways suggests that biological variables have a role in the etiology of postpartum psychiatric illnesses. It is essential to expand the field of study to encompass relations to various organs that are known to be altered by pregnancy to create novel preventative and curative measures for these illnesses (Zhang et al., 2023b). In considering this, there is growing data that suggests the immune system is a plausible candidate for the development and maintenance of postpartum mental disorders. Immune changes occur during pregnancy and childbirth, and depression and other mental health issues outside of the peripartum phase have been linked to deregulated inflammatory responses. Therefore, it makes sense that peripartum mood problems could be influenced by a similar process (Alesci et al., 2022).

This study aims to investigate the complex hormonal-immune relationships that impact the health of mothers both during pregnancy and after giving birth. It seeks to comprehend how immune adaptations and endocrine controls at the feto-maternal interface contribute to the success of pregnancy. The study also assesses therapy modalities for postpartum hormonal alterations, such as hormonal, pharmacological, and non-pharmacological therapies. This review aims to elucidate the bidirectional interactions between hormonal fluctuations and immune reconstitution in the postpartum period, with a specific focus on their role in the pathogenesis of postpartum mood disorders (PPD, PPA, and PP).

## Hormonal changes in the postpartum period

**FIGURE 2:**
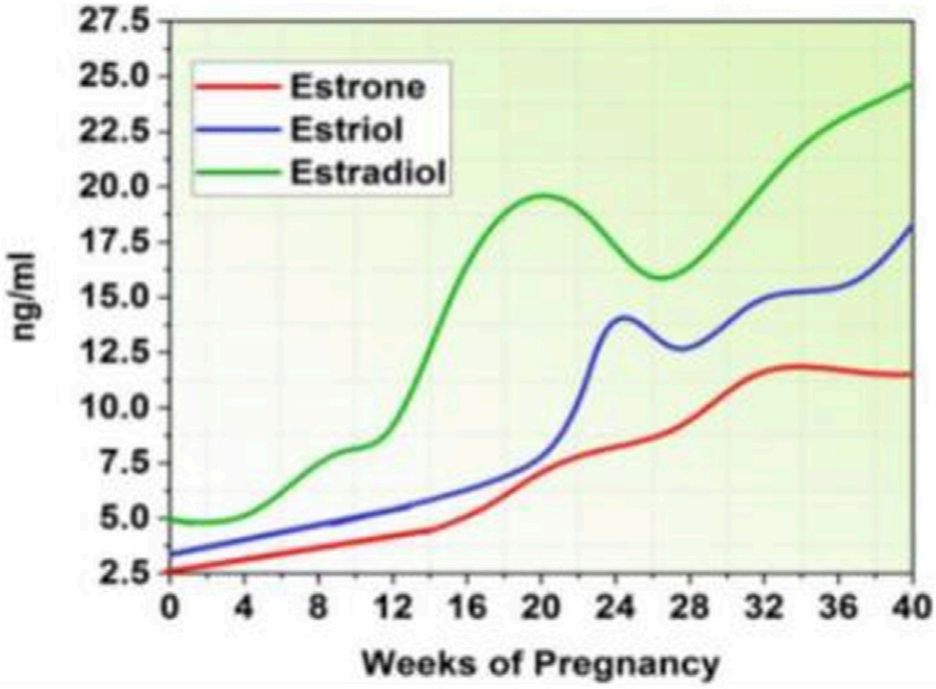
Estrogen level.

The invention of these hormones by the placenta is largely responsible for the steady increase in levels of progesterone and estrogens during pregnancy. Estrogen and progesterone levels plummet following the removal of the placenta during birth, but they regain their pregravid levels by the fifth postpartum day (Molinet Coll et al., 2022). Human chorionic gonadotrophin, cortisol, and beta-endorphin levels similarly increase during pregnancy, peaking close to term and then falling upon delivery (Konjevod et al., 2023). Figure 2 represents the increased estrogen levels during pregnancy (Pestana et al., 2023).

**FIGURE 3:**
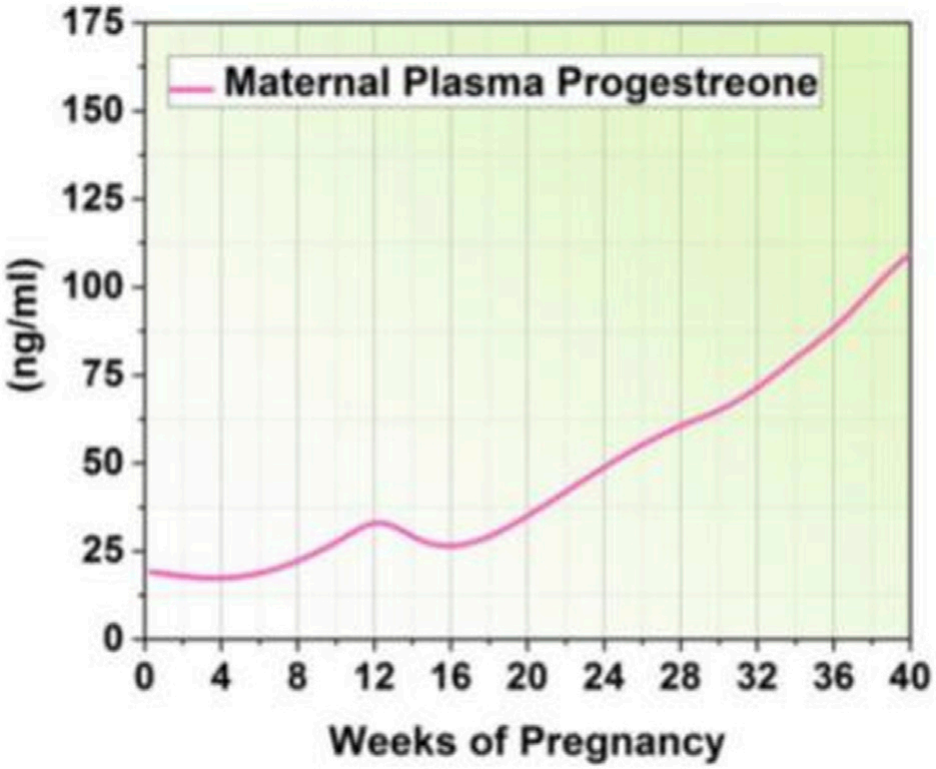
Progesterone level.

Total Triodothyronine (T3) and Thyroxine (T4) levels decrease after delivery due to the decrease in thyroid-binding globulin, whereas free T3 and T4 levels stay mostly unchanged (Wieczorek et al., 2023). By stimulating pituitary lactotrophic cells with the hormone oxytocin, breastfeeding maintains high levels of prolactin. However, prolactin levels gradually revert to pregravid levels, even in nursing mothers (Dye et al., 2022). Table 2 highlights the possible correlations between postpartum psychological fitness disorder, including anxiety and depression and the hormonal patterns that occur throughout pregnancy and the postpartum period. Figure 3 shows an increase in progesterone levels during pregnancy.

**TABLE 2:** The relationships between hormonal changes and postpartum mental health.

Hormone	Pattern during pregnancy	Postpartum pattern	Associated effects on postpartum mental health
Cortisol	The placental corticotrophin-releasing hormone causes it to peak in the latter stages of pregnancy	After birth, it declines significantly	Most studies found no significant association with PPD
Thyroid hormones	Thyroid function impairment prevalence increased somewhat after giving birth	Up to 7% of women experience thyroid issues	Some women’s PPD is connected to thyroid dysfunction; in 38% of cases, treating thyroid problems reduces depression
Prolactin	Reaches about 140 ng/mL in the latter stages of pregnancy	After giving birth, non lactating women have declined, while nursing women experience stabilization	Pathologic hyperprolactinemia in nonpregnant women is associated with anger, anxiety, and depression
Oxytocin	Increases significantly during lactation and delivery	Varies according to nursing activities	Though its connection to PPD is indeed unknown, it could have an impact on bonding and mood
Vasopressin	Elevated while pregnant	Plasma levels are unchanged but lower in postpartum urine	Controls blood pressure and electrolyte balance; unclear role in PPD
Estrogen	Increases during pregnancy by about 100 times	Abruptly declines after giving birth	Although there is no discernible difference in the variations in estradiol between women who are sad and those who are not, a sudden reduction could render PPD worse

### Hormones of cortisol

The synthesis of corticotropin-releasing hormone by the placenta causes cortisol levels to peak in late pregnancy and then sharply decline at delivery. Urinary-free cortisol, also known as plasma cortisol, has not been related to PPD in several studies (Borgsted et al., 2022). A study that found a beneficial association across early levels of serum cortisol, measured 6 weeks after giving birth, and the level of dysphoria in women was complicated by the fact that breastfeeding timing and the inability to control stressful life events can cause cortisol levels to rise or fall, respectively (Baattaiah et al., 2023).

### Hormones of the thyroid

Following childbirth, there is a modest increase in the prevalence of impaired thyroid function. Thyroid dysfunction affects women in the 6 months after giving birth, compared to the general population. Thyroid dysfunction could contribute to PPD in a subset of women, even if it has not been found in the majority of these women (Cheng et al., 2022). Seven percent of the 303 pregnant euthyroid women in the prospective trial went on to develop postpartum thyroid problems. Treatment for thyroid dysfunction addressed the depression that was found in 38% of these mothers. Weight gain, cold sensitivity, and tiredness are indications of hypothyroidism; thus, measuring thyroid function is a crucial component of assessing PPD in women with these symptoms (Luo et al., 2023).

### The pituitary hormones

In late pregnancy, prolactin levels increase from pregravid levels and in nonlactating women, they decrease in the 3 weeks following birth. Prolactin levels in mothers rise for a few months before falling to levels seen before pregnancy (Barooj-Kiakalaee et al., 2022). There has been no research done on the relationship between PPD and the posterior pituitary chemicals oxytocin and vasopressin, which change in amounts throughout the postpartum phase. During labor, oxytocin stimulates uterine muscle contraction and encourages the flow of breast milk (Yadav et al., 2023). It also rises dramatically during delivery and throughout breastfeeding. A measure of mood state, vasopressin, which controls blood pressure and electrolyte balance, was shown to be lower in postpartum women’s urine but not in their plasma when compared to nonpuerperal women (Avila-García and Figueroa-Damián, 2023).

### Steroids of gonadal

The physiologically active forms of estrogen, estradiol, and estriol are produced by the placenta and increase by times throughout pregnancy. Estriol is produced in large quantities during pregnancy because it is a byproduct of the fetal liver’s metabolic activities (Barinov et al., 2022). Estradiol has been shown in animal experiments to improve neurotransmitter function by increasing serotonin production and decreasing its breakdown (Kim et al., 2022). Therefore, PPD could be exacerbated by the sudden drop in *estradiol* levels after delivery (Al Beloushi et al., 2024). The degree of alteration in total oestradiol or complimentary *oestriol* since delayed pregnancy, however, did not differ significantly between depressed and no depressed women, according to a maximum pregnant lady (Yilma et al., 2023).

## Inflammatory reactions and immune reconstitution

Pregnancy maintenance depends on an immunosuppressive state marked by anti-inflammatory reactions. While Th1 cytokines, which could be harmful to the developing child, are *modulated* throughout pregnancy, Th2 and Th3 responses, which promote pregnancy, are increased. During pregnancy, maternal hormones, such as progesterone, cortisol, norepinephrine, and 1, 25-dihydroxyvitamin are important in regulating immunological responses (Escudero et al., 2023). At the maternal-fetal border, there is also a change in restricted immunoreactivity towards Th2. A normal or even increased proinflammatory response could be linked to the postpartum phase change in the mother’s immune repertoire toward Th1 (Schaffenburg et al., 2022). Table 3 illustrates the pregnancy-related humoral and cellular immunological responses. The shift from Th2-dominant immunosuppression during pregnancy to Th1-mediated proinflammatory responses postpartum may exacerbate neuroinflammation, thereby contributing to the dysregulation of neurotransmitters (e.g., serotonin and dopamine) implicated in PPD and PPA. This immunological transition underscores the need to explore immune biomarkers as predictors of postpartum mental health outcomes.

**TABLE 3:** Immunological reactions of the humoral and cellular systems during pregnancy.

Variable	Reactions
Humoral immunity Harmonic supplements	Elevated amounts of complement regulatory proteins, including membrane cofactor protein (CD46), decay accelerating factor (CD55), and CD59, as well as elevated levels of C3, C4, and Clq
Reactants in the innate acute phase	An enhancement in acute-phasereactants, including ceruloplasmin and fibrinogen
Cellular immunity Granulocytes and innate monocytes	Elevated levels, improved respiratory burst activity and phagocytosis, and surface expression
Spontaneous NK cells	Progesterone-induced blocking factor and downregulate cytotoxic activity; IFN-g production is reduced

### Immune compensation and autoimmune disorders in the postpartum period

For instance, compared to other women, pregnant women had a 70% lower risk of developing rheumatoid arthritis symptoms (Gottschling et al., 2024). However, there is a significantly higher chance of getting it during the postpartum phase, especially in the first three months. The relapse rate in women with multiple sclerosis falls during pregnancy, rises during the first three months of the postpartum phase, and then falls back to the pre-pregnancy rate (Shmakov et al., 2022).

Other autoimmune conditions, such as Hashimoto thyroiditis and Graves’s disease, also resolve on their own during pregnancy before getting worse after giving birth (Vasconcelos et al., 2022). Three to six months after giving birth, 70% of moms with positive thyroid stimulating antibody screening test findings experienced either temporary or permanent postpartum Graves’s disease. Additionally, postpartum cases of autoimmune myocarditis, idiopathic polymyositis, hemolytic uremic syndrome, antiphospholipid antibody syndrome, and sarcoidosis have been reported (Zaigham et al., 2022).

### Causes of infections and types

Women throughout their postpartum period were afflicted with a variety of infections, including bacterial, fungal, and viral infections (Tesfaye et al., 2022).

#### Viruses

##### Herpes and hepatitis virus

It is a well-known virus infection with rapid syndrome sequence due to immunological reconstitution-mediatedliver damage that *occurs* inHIV-infected individuals starting strong antiretroviral regimens (Collins et al., 2022). During pregnancy, HCV RNA levels dramatically dropped and Alanine Aminotransferase (ALT)levels returned to normal; the third trimester showed the lowest viral load (Engler-Chiurazzi et al., 2022). One month after delivery, there was a 120-fold increase in ALT levels and a sudden rise in Hepatitis C Virus (HCV) RNA levels that coincided with a hepatitis flare (Burgos-Gamez et al., 2023). Three months after birth, the ALT level returned to normal and the HCV RNA level dropped. As has been recommended for patients with HIV co infection starting, it is wise to monitor liver enzymes in postpartum women who are carrier’s virus. Although it could seem counterintuitive, patients with HIV-HCV co-infection have also been seen to experience an increase in viral load in tandem with immunological recovery (Çankaya and Atas, 2022). There have been case reports of postpartum endometritis brought on by a herpes simplex virus infection, which left the mother with serious aftereffects. Two neonates died as a result of transmission to the newborn, which caused a widespread infection (Taylor, 2023).

#### Bacteria

##### Mycobacterium leprae

The two forms of leprosy disease manifestation are the *lepromatous form*, which is linked to a high bacillary load and insufficient cellular responses. Overt leprosy could appear in women infected with *M. leprae* during the postpartum phase. Furthermore, the disease has started during lactation in as many as 6% of pregnant women’s leprosy cases. Similarly, there is a 50% chance that pregnant patients undergoing leprosy therapy would develop *lepromatous leprosy*. It has been suggested that women who have recovered from leprosy be monitored for the emergence of exchange reaction or signs of spirit injury; if these reactions appear, high-dose *corticosteroid* treatment should be taken into consideration (Grilo et al., 2022).

##### Mycobacterium tuberculosis

Pregnancy has been associated with a decrease in cell-mediated immunity, as seen by the gradual weakening of lymphocyte responsiveness to pure protein antigens. These findings imply that the quick reconstitution of pathogen-specific cellular immune responses could be linked to the worsening of latent *tuberculosis* during the postpartum phase. In those who have already caught *M. tuberculosis*, cell *responses* for crucial effectively stop reactivation at the locations of the original and *lymphohematogenous* metastatic foci. *Granulomatous* lesions caused by TB in the postpartum phase show a lot of inflammatory cells but not many bacteria, indicating the host’s attempts to control the infection.

#### Fungi

##### Among various mycoses, *Coccidioides immitis*

Pregnancy has been strongly associated with distributed disease, particularly in the postpartum phase, even though it does not raise the risk of *coccidioidomycosis*. The mortality rate for 29 *coccidioidomycosis* cases that happened in the pregnancy was 55%, and the transience rate for 7 cases that happened in the postpartum phase was 29%. Ten instances of *coccidioidomycosis* were found in 47,120 pregnancies among pregnant women in an endemic area. According to a different report, only two out often pregnant women experienced disseminated disease, and both of them fell *unwell* in the postpartum phase. Patients with a history of *coccidioidal* immunological reactivity decline in cell-mediated immunity during pregnancy.

##### Cryptococcus neoformans

In cryptococcosis patients, a Th2 reaction makes infection easier to establish. A similar tendency has been documented in case reports of pregnant women whose latent *cryptococcal* infection abruptly deteriorated during the postpartum phase, while rare, mother-to-child transmission of *cryptococcal* infection has been documented. According to an analysis of pregnancy-related cryptococcosis, the third trimester or the postpartum phase accounted for 45% of cases. The infection most likely happened during pregnancy, and since the immune system was stimulated and Immune Reconstitution Syndrome (IRS) was formed during the postpartum phase, no antifungal medication was required (Garapati et al., 2023). Table 4 identifies several pathogens that, as a result of immune system alterations, can recur or worsen during the postpartum phase, along with the clinical signs that could accompany these infection.

**TABLE 4:** Pathogens in the postpartum period with a higher risk of reactivation and associated clinical symptoms.

Pathogens	Clinical signs and symptoms
Viruses	
*Herpes virus*	Endometritis is caused by the Herpes Simplex Virus and increased cytomegalovirus excretion
*Hepatitis virus*	Aminotransferases and HCV RNA levels are higher in chronic HCV carriers
Bacteria	
*Mycobacterium leprae*	Neuritis and skin lesions brought on by tuberculoid leprosy
*Mycobacterium tuberculosis*	Meningitis, CNS lesions, osteoarticular infections, and pulmonary infiltrates
Fungi	
*Coccidioides immitis*	Widespread infection, especially in the third trimester and after giving birth
*Cryptococcus neoformans*	Soft-tissue or osteoarticular infection, lung nodules and/or infiltrates, meningitis, and CNS lesions

## Hormonal-immune interactions’ effects on maternal health

The dynamic interplay between the mother, placenta, and semi-allogeneic fetus makes maternal immunological tolerance one of the most important factors of a successful pregnancy. The pregnancy tolerogenic state is caused by tissue, cellular and molecular immune system adaptations in the mother. Villous and extravillous trophoblasts (EVTs) that express specific major histocompatibility complex (MHC) molecules control immune functions at the feto-maternal interface.

### Immunological adaptations are endocrinely regulated

Immunological tolerance at the matemal-fetal interface depends on endocrine-driven mechanisms and the interaction of lymphocytes and antigen-presenting cells (APCs). Cytokine-producing CD56brightCD16- uNK leukocytes, which are the most common (50%–70%) during the first trimester of pregnancy, help with embryo implantation and trophoblast invasion and differentiation (Kazemi et al., 2023). Among other aspects, uNK cell formation, proliferation, and the synthesis of chemicals that promote embryonic growth are influenced by the activation of the *estrogen receptor β* and HLAG/ILT2.

HLA-G/ILT2-ILT4 signaling in the uterus stimulates the development of M2 macrophages with scavenger receptors and anti-inflammatory characteristics. By producing IL-10, the regulatory responses were removed. Subpopulations of lymphocytes mediate immunological tolerance toward fetal *alloantigens* and coordinate cellular responses against exogenous antigens (Kuravska et al., 2022). Table 5 represents the immune and hormone responses during pregnancy.

**TABLE 5:** Effects of the immune system and hormones during pregnancy.

Hormone/Immune factor	Immune response	Effect on maternal health
HLA-G/ILT2 signaling	Stimulates the implantation of immune cells (uNK)	Enhances immunological tolerance and promotes a successful pregnancy
Estrogen (ER β activation)	Promotes the formation of immunological cells	Early pregnancy and trophoblast invasion are encouraged
M2 Macrophages	Tissue regeneration and anti-inflammatory	Aids in pregnancy and the healing process after giving birth
Regulatory T and B cells	Mediates fetal alloantigen tolerance	Reduces the likelihood of immunological rejection by balancing immune responses
T-cell balance (Th1, Th2, Treg, Th17)	Increases Tregs and decreases inflammatory T-cells	Keeps the immune system from becoming overactive, guaranteeing a safe pregnancy
Cytokines (IL-10, IL-6)	Controls the activity of trophocytes and immunological responses	Keeps the immune system balanced, promoting a healthy pregnancy

### Hormonal-immune axis and postpartum psychiatric disorders

“Emerging evidence suggests that cytokine imbalances (e.g., elevated IL-6 and TNF-α) coupled with estrogen depletion may impair hippocampal neurogenesis, a mechanism implicated in PPD (Smith et al., 2021). Conversely, progesterone’s anti-inflammatory properties could mitigate neuroinflammation, highlighting potential therapeutic targets for PPA and PP.”

## Effective approaches in therapy for recovering from postpartum hormonal changes

**FIGURE 4:**
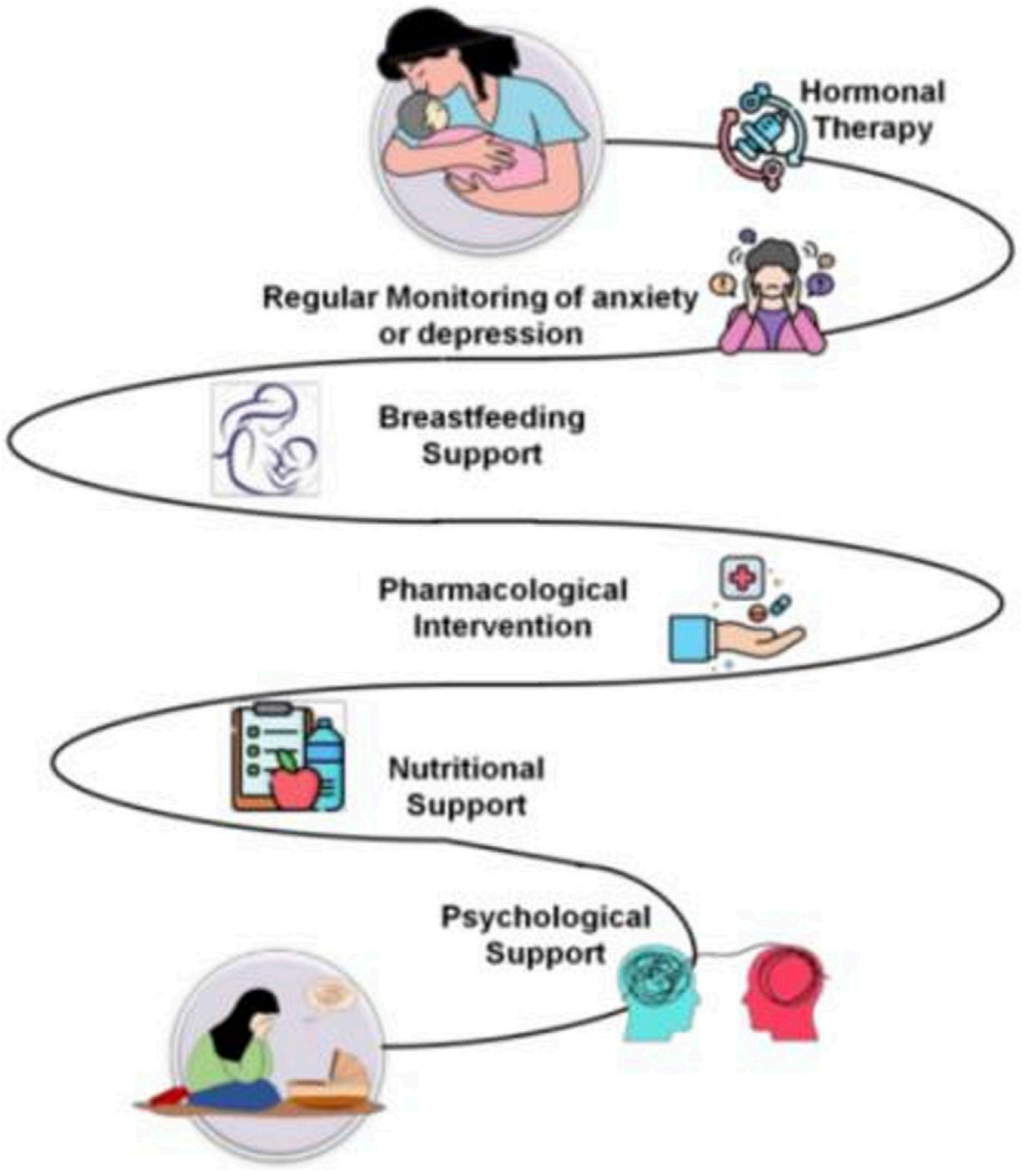
Therapy approaches.

Therapy is defined by comprising at least four symptoms: higher or lower hunger, difficulty sleeping, psychomotor disorder or delay, feelings of inadequacy, with at least 2 weeks of continually low mood. During the postpartum phase, these standards are the same as they were in earlier periods. Figure 4 represents the types of therapy for recovering from the hormonal changes on postpartum. “The interplay between estrogen withdrawal and Th1 cytokine surge may synergistically disrupt hypothalamic-pituitary-adrenal (HPA) axis regulation, amplifying vulnerability to PPD. Future studies should prioritize longitudinal assessments of hormonal-immune crosstalk in postpartum mood disorders, particularly through integrated omics approaches (e.g., cytokine profiling and steroid hormone assays).”

### Hormone therapy

It has been proposed that one of the factors causing PPD in certain women is the significant drop in maternal progesterone and estrogen levels that occurs after delivery. Estrogen not only enhances neurotransmitter function but also changes the hypophyseal-pituitary axis, reduces oxidative stress, and encourages neuronal growth and survival (Naghshineh et al., 2023). In an effort to replicate the hormonal shifts that take place around the time of delivery, this was done. Initial results from using estrogen to treat PPD have been favorable (Ye et al., 2023).

### Regular evaluation for anxiety or postpartum depression

The significance of early and precise identification and therapy for depressive disorders during or throughout pregnancy has come into greater focus in an effort to lessen the severe negative impacts of PPD.

Diagnoses of PPD could be more difficult because of the typical substantial and expressive difficulty of the new maternity period, such as altered energy and appetite, lack of sleep, and heightened anxiety over the baby (Dessì et al., 2024).

### Breastfeeding support

The potential impact of depressive drugs on nursing worries a lot of women and professionals. Neonates and early newboms are especially vulnerable to the negative effects of some medications due to their growing neural systems, underdeveloped hepatic and renal systems, and undeveloped blood-brain barriers. Several specialists have suggested nonpharmacologic therapy methods wherever possible, especially for mild to moderate depression, due to the paucity of information regarding the effects of antidepressant medication in breast milk (Jyotsna et al., 2023).

### Pharmacological interventions

PPD could be a subtype of major depression that reacts similarly to antidepressant drugs, according to a modest but increasing body of research. The pharmacological action of PPD increases anxieties about postnatal metabolic variations, the infant’s contact to medication in breast milk, the influence of unhappiness and handling on the miserable mother’s capacity to care for a newborn, and the dishonor related with being branded a bad mother for demanding medicine (Rupanagunta et al., 2023).

### Psychologicalsupport

Many mothers with PPD are reluctant to use antidepressants and often choose for psychological therapy instead, due to worries about potential side effects or about exposing their infant to medicine through breast milk. The evidence that is currently available supports the use of emotional therapies, even if research on nonpharmacologic treatments for PPD is yet in its early phases (Kleiman, 2022). Both psychological and psychosocial therapy are effective in reducing depression and are a significant alternative for treating PPD, according to a study often randomized controlled trials. These therapies include non-directive counselling, cognitive behavioral therapy, interpersonal therapy, and support from partners and peers (Gunduz-Bruce et al., 2022).

### Nutritional support

Omega-3 fatty acids have gathered special kindness in the treatment of perinatal unhappiness due to their well-established wellbeing compensations for anxious and postpartum women, as well as some indication of their positive belongings on mood in the general people. The development of a fetus’s central nervous system depends on omega-3 fatty acids present in fish oils. This process is aided by the mother’s omega-3 fatty acid depletion during pregnancy (Xie et al., 2024).

## Discussion

Examining the hormonal-immune interactions that occur throughout pregnancy and their impact on maternal health, especially postpartum recuperation, is the aim of this investigation. Immunological cells, hormones, and the feto-matermal interface work intricately to control maternal immunological tolerance, which is necessary for a healthy pregnancy. Together, immune cells like macrophages, regulatory T and B cells, and Natural Killer (NK) cells support implantation, trophoblast invasion, and angiogenesis while preserving immunological tolerance for the fetus. By altering neurotransmitter function and brain health, hormonalchanges, specifically the drop inestrogen and progesterone following delivery, contribute to PPD. Estrogen supplements and hormone treatment have shown efficacy in treating PPD, but they must be taken carefully because of the risks of thrombosis and interfering with breastfeeding. Furthermore, dietary assistance, especially omega-3 fatty acids, helps to enhance mood control in the postpartum phase. Therefore, to ensure the health and wellbeing of mothers during their recuperation, a multifaceted strategy incorporating hormonal, psychological, and dietary therapies is essential.

### Conclusion

The principle of the examination is to investigate the hormonal-immune interactions that take place throughout pregnancy and their effects on maternal health, particularly postpartum recovery. Overall, healthy pregnancy and the development of the fetus depend on the hormonal-immune interactions that control the mother’s immunological tolerance during pregnancy. PPD is largely caused by hormonal changes that occur after delivery, especially the decrease in progesterone and estrogen, which calls for cautious control. Nonpharmacological therapies like psychological support, breastfeeding assistance, and nutritional interventions like omega-3 fatty acids are just as important for promoting maternal recovery as hormone therapy and pharmaceutical treatments, which offer promising avenues for alleviating postpartum mood disorders. To address the various requirements of mothers during the postpartum period and to guarantee their general wellbeing, a thorough and customized approach that combines medical and psychological assistance is necessary. To prevent and cure PPD, future studies should concentrate on investigating individualized hormonal and immune-based treatments. The long-term impacts of combining pharmaceutical and non-pharmacological interventions on maternal health also require more research.
